# Sex-Dependent Cardiac Responses to β3-Adrenergic Receptor Activation in a Murine Model of Heart Failure with Preserved Ejection Fraction

**DOI:** 10.3390/biomedicines14071633

**Published:** 2026-07-20

**Authors:** Sara-Ève Thibodeau, Élisabeth Walsh-Wilkinson, Emylie-Ann Labbé, Diwaba Carmel Teou, Marie-Lune Legros, Jacques Couet

**Affiliations:** 1 Département de Médecine, Faculté de Médecine, Université Laval, Québec City, QC G1V 0A6, Canada; sara-eve.thibodeau@criucpq.ulaval.ca (S.-È.T.); elisabeth.walsh-wilkinson.1@ulaval.ca (É.W.-W.); emylie-ann.labbe.1@ulaval.ca (E.-A.L.); diwaba-carmel.teou.1@ulaval.ca (D.C.T.); marie-lune.legros.1@ulaval.ca (M.-L.L.); 2 Groupe de Recherche sur les Valvulopathies, Centre de Recherche de l’Institut Universitaire de Cardiologie et de Pneumologie de Québec, Université Laval, Québec City, QC G1V 4G5, Canada; 3 Centre de Recherche de l’Institut Universitaire de Cardiologie et de Pneumologie de Québec, Université Laval, Québec City, QC G1V 4G5, Canada

**Keywords:** β3-adrenergic receptor, mirabegron, HFpEF, brown adipose tissue, cardiac remodelling, sex differences

## Abstract

**Background:** Brown adipose tissue (BAT) is increasingly recognized as an endocrine organ that releases bioactive factors (batokines) with cardioprotective properties. Activation of BAT is primarily mediated by the β3-adrenergic receptor (β3-AR). Here, we investigated whether pharmacological activation of β3-AR using mirabegron modulates cardiac remodelling in a murine model of heart failure with preserved ejection fraction (HFpEF) induced by metabolic and hypertensive stress (MHS). **Methods:** Male and female C57BL/6J mice were exposed to MHS (angiotensin II + high-fat diet) for 28 days, with or without mirabegron treatment (2 mg/kg/day). Cardiac structure and function were assessed by echocardiography, and molecular and histological analyses were performed on cardiac and BATs. **Results:** Mirabegron attenuated several features of cardiac remodelling in males, including cardiac hypertrophy, left atrial enlargement, and left ventricular dilation. These effects were associated with reduced expression of genes related to hypertrophy and fibrosis. In contrast, it was shown that females exhibited a less pronounced response pattern. BAT mass and thermogenic gene expression (Ucp1) increased more markedly in males than in females, suggesting differential BAT responsiveness between sexes. **Conclusions:** β3-AR activation is associated with sex-dependent cardiac responses in this HFpEF model, with more pronounced protective effects in males. These findings are consistent with a potential contribution of BAT activation to cardiac remodelling, although causality was not directly demonstrated in the present study.

## 1. Introduction

Heart failure with preserved ejection fraction (HFpEF) is a complex and heterogeneous clinical syndrome driven by multiple comorbidities, including hypertension, obesity, diabetes, kidney disease, atrial fibrillation, ageing, and sex-related factors such as menopause [1]. HFpEF is associated with a high burden of morbidity and mortality, with up to 80% of patients experiencing hospitalization and nearly 50% dying within five years of diagnosis [2]. Despite its growing prevalence, effective therapeutic strategies remain limited.

To better understand the pathophysiology of HFpEF, several preclinical models have been developed. Notably, the combination of nitric oxide synthase inhibition with a high-fat diet (HFD) has been widely used to reproduce key features of the disease [3]. This “multi-hit” approach has since been extended to incorporate additional cardiovascular and metabolic stressors [4,5,6,7]. These models typically recapitulate hallmark features of HFpEF, including concentric left ventricular (LV) remodelling, cardiac hypertrophy, diastolic dysfunction, myocardial fibrosis, and preserved ejection fraction.

We recently developed a murine HFpEF model based on combined metabolic and hypertensive stress (MHS), induced by angiotensin II (AngII) infusion and HFD feeding [8]. This model reliably reproduces several key characteristics of HFpEF, including LV hypertrophy, left atrial enlargement, impaired exercise capacity, and myocardial remodelling in both male and female mice [8,9]. Importantly, this model also revealed sex-dependent differences in disease progression, with females generally exhibiting a less severe phenotype [7,8,9].

HFpEF is increasingly recognized as a systemic disorder involving multiple organs and tissues beyond the heart. Notably, adipose tissue has emerged as a key contributor to disease pathophysiology. Given that a large proportion of HFpEF patients are obese, adipose tissue-derived factors are likely involved in modulating cardiac function and remodelling. Our previous work demonstrated that circulating microRNA profiles are significantly altered in this model, with adipose tissue and liver representing major contributors to circulating miRNAs [10].

Brown adipose tissue (BAT), traditionally viewed as a thermogenic organ, is now recognized as an active endocrine tissue capable of secreting bioactive molecules, termed batokines, that influence distant organs, including the cardiovascular system [11,12]. BAT activity is tightly regulated by the β3-adrenergic receptor (β3-AR), which is highly expressed in this tissue. Activation of β3-AR signalling has been shown to confer cardioprotective effects in various pathological contexts, including models of hypertension, ischemia–reperfusion injury, and cardiac remodelling [11,12,13,14].

In addition to environmental stimuli such as cold exposure, BAT can be pharmacologically activated with selective β3-AR agonists, including mirabegron. Mirabegron has been shown to reduce cardiac remodelling in male mice subjected to AngII-induced stress [13,14]. However, its effects in HFpEF models, particularly across both sexes, remain poorly characterized.

Interestingly, we previously showed that environmental temperature modulates the HFpEF phenotype in mice. Housing at thermoneutrality (30 °C), where BAT activity is minimal, attenuated disease severity, whereas cold exposure exacerbated cardiac dysfunction [15]. These findings highlight the complex relationship between BAT activation and cardiac remodelling, suggesting that the context and mode of activation may be critical determinants of outcome.

In the present study, we investigated whether pharmacological activation of β3-AR using mirabegron modulates cardiac remodelling in our MHS-induced HFpEF model. Given the known sex differences in both HFpEF and BAT physiology, we specifically examined whether these effects differ between male and female mice.

We hypothesized that β3-AR activation through mirabegron would attenuate cardiac remodelling in HFpEF by promoting BAT activation, and that this response would differ between sexes.

## 2. Materials and Methods

### 2.1. Animals

C57BL/6J male and female mice (7 weeks old) were purchased from The Jackson Laboratory (Bar Harbor, ME, USA). Animals were housed 3–4 per cage under controlled conditions (12 h light/dark cycle, 22 °C) with ad libitum access to food and water.

All procedures were approved by the Université Laval Animal Protection Committee and were conducted in accordance with the Canadian Council on Animal Care (CCAC) guidelines (protocol #2023-1249) and ARRIVE guidelines.

Mice were randomly assigned to experimental groups (n = 7–8 per group). Health status and behaviour were monitored daily by trained personnel, and body weight was recorded weekly. Three male mice died in the MHS group during the protocol.

**Figure 1:**
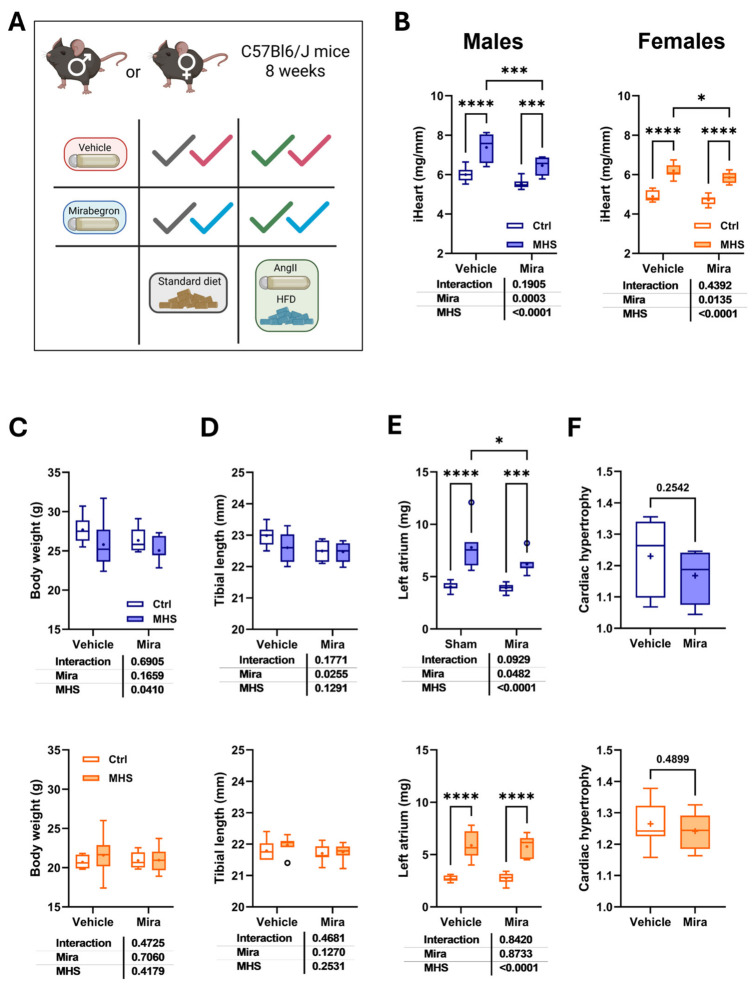
Effects of mirabegron on the mouse body and cardiac morphology with or without MHS. (**A**) Schematic of the experimental design (created in BioRender. Couet, J. (2025) https://BioRender.com/wmcm34z (accessed on 30 September 2025)). Eight-week-old C57BL/6J male and female mice were divided into four groups for 28 days: control mice on a standard diet receiving the vehicle; MHS mice treated with the vehicle; control mice treated with mirabegron (Mira); and MHS mice receiving Mira. (**B**) Indexed heart weight for tibial length (iHeart). Males: blue and left panel. Females: orange and right panel. (**C**) Body weight. Males: blue and upper panel. Females: orange and lower panel. (**D**) Tibial length. (**E**) Left atrial weight. (**F**) Cardiac hypertrophy, expressed as the ratio of heart weight in MHS mice to the average heart weight of the corresponding control group. Results are shown as mean ± standard error of the mean (SEM)—two-way ANOVA followed by Fisher’s LSD test. Two-way ANOVA results are shown below each graph, including the interaction (MHS × Mira). * *p* < 0.05, *** *p* < 0.001, and **** *p* < 0.0001 between indicated groups (n = 7–8 mice/group).

The experimental design is illustrated in Figure 1A.

### 2.2. Metabolic and Hypertensive Stress (MHS)

Mice were subjected to a combined metabolic and hypertensive stress consisting of continuous angiotensin II (AngII) infusion and a high-fat diet (HFD), as previously described [8].

AngII (1.5 mg/kg/day; Sigma-Aldrich, Mississauga, Ont, Canada) was delivered via subcutaneously implanted osmotic minipumps (Alzet model #1004, Campbell, CA, USA) for 28 days. Mice were simultaneously fed a high-fat diet (60% kcal from fat; Research Diets, D12492, New Brunswick, NJ, USA).

For β3-adrenergic receptor activation, mirabegron (Toronto Research Chemicals, Toronto, Canada) was administered via osmotic minipumps (Alzet #1004) at 2 mg/kg/day for 28 days. Mirabegron was dissolved in a vehicle solution containing DMSO and PEG400.

### 2.3. Echocardiography

Transthoracic echocardiography was performed under isoflurane anesthesia as previously described [8,16]. Cardiac dimensions and functional parameters were obtained using standard M-mode and two-dimensional imaging. The person imaging the animals was blinded to the groups.

### 2.4. RNA Isolation and Quantitative Real-Time PCR

Total RNA was isolated from left ventricular (LV) tissue using the TRIzol method (Invitrogen, Burlington, ON, Canada), as previously described [8]. RNA concentration and purity were assessed spectrophotometrically.

cDNA was synthesized using standard reverse transcription protocols, and quantitative real-time PCR (qPCR) was performed to assess gene expression levels.

Cyclophilin A (Ppia) was used as the housekeeping gene for cardiac tissue, while ribosomal protein L13 (Rpl13) was used for BAT analyses. Relative gene expression was calculated using the ΔΔCt method. Primer sequences are listed in Table 1.

### 2.5. Myocardial Fibrosis

Left ventricular tissue sections (10 µm) were stained with Picrosirius Red to assess interstitial fibrosis. Fibrosis was quantified as the percentage of fibrotic area relative to total tissue area using the formula: (% fibrosis)/(% fibrosis + % tissue) × 100. Perivascular fibrosis was excluded from the analysis. Investigators performing histological and morphometric analyses were blinded to treatment allocation.

### 2.6. Cardiomyocyte Cross-Sectional Area

Cardiomyocyte size was assessed using wheat germ agglutinin (WGA)-FITC staining (Sigma-Aldrich), as previously described [17]. Cross-sectional area (CSA) was measured on LV sections using fluorescence microscopy.

### 2.7. BAT Histology and Immunohistochemistry

Brown adipose tissue (BAT) samples were fixed in 4% paraformaldehyde for 24 h, transferred to 70% ethanol, and paraffin-embedded. Sections (4 µm) were stained with hematoxylin and eosin for morphological analysis. UCP1 protein expression was assessed by immunohistochemistry using a polyclonal rabbit anti-UCP1 antibody (1:1000 dilution) in Tris-buffered saline containing Triton X-100 and 5% milk, followed by an HRP-conjugated secondary antibody.

### 2.8. Statistical Analysis

Data are presented as mean ± standard error of the mean (SEM). Outliers were identified using the ROUT method (Q = 1%) in GraphPad Prism (v10.6). Normality was assumed based on sample size and distribution consistency.

Comparisons between two groups were performed using unpaired Student’s *t*-tests. For multiple group comparisons, two-way ANOVA followed by Fisher’s least significant difference (LSD) post hoc test was used. A *p*-value < 0.05 was considered statistically significant. For variables analyzed using two-way ANOVA, interaction terms were evaluated and are reported in the corresponding figures and tables.

To facilitate visualization of male–female response patterns, direct sex comparisons for selected cardiac and BAT-related endpoints are presented in Supplementary Figures S1–S4.

## 3. Results

### 3.1. Effects of Mirabegron on Body and Cardiac Growth

As illustrated in Figure 1A, C57BL/6J male and female mice were subjected to metabolic and hypertensive stress (MHS) for four weeks through combined angiotensin II (AngII) infusion and high-fat diet (HFD), with or without mirabegron treatment. Control mice receiving either vehicle or mirabegron were studied in parallel.

Mirabegron treatment reduced indexed heart weight (normalized to tibial length), indicating a partial attenuation of cardiac hypertrophy (Figure 1B). In MHS mice, mirabegron also resulted in smaller hearts despite the hypertrophic stimulus. Body weight remained unchanged with mirabegron treatment in both sexes; however, tibial length was significantly reduced in males but not in females, suggesting a sex-dependent pattern on body growth (Figure 1C,D).

As previously reported [8], MHS induced left atrial (LA) enlargement in both sexes. This effect was attenuated in males treated with mirabegron, whereas no significant change was observed in females (Figure 1E). When cardiac hypertrophy was normalized to respective control groups, no significant differences were detected between mirabegron-treated and untreated mice (Figure 1F), suggesting that the overall hypertrophic response was not completely prevented.

### 3.2. Effects of Mirabegron on Cardiac Structure and Function

**Figure 2:**
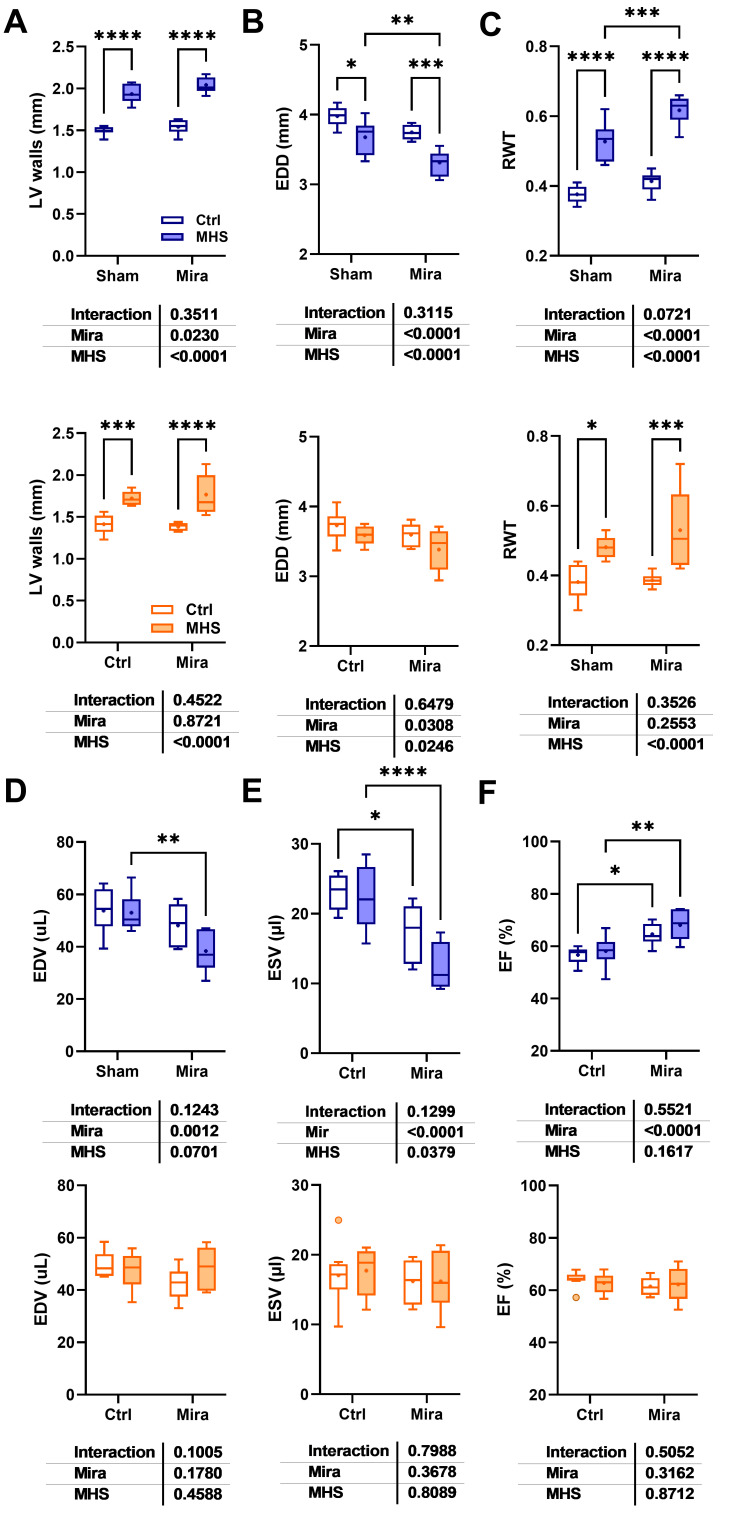
Mirabegron (Mira) reduces left ventricular volumes and increases EF in males but not females. (**A**) Diastolic LV wall thickness (posterior + septal). Males: blue and upper panel. Females: orange and the bottom panel. (**B**) End-diastolic LV diameter (EDD). (**C**) Relative wall thickness (RWT; LV walls/EDD). (**D**) End-diastolic volume (EDV). (**E**) End-systolic volume (ESV) and (**F**) LV ejection fraction (EF; (EDV − ESV)/EDV ∗ 100). Results are expressed as mean ± standard error of the mean (SEM)—two-way ANOVA followed by Fisher’s LSD test. The results of the two-way ANOVA are presented below the graph for each variable and the interaction (MHS × Mira). * *p* < 0.05, ** *p* < 0.01, *** *p* < 0.001 and **** *p* < 0.0001 between indicated groups (n = 7–8 mice/group).

Cardiac structural and functional changes are further detailed in Figure 2. As expected, MHS induced concentric left ventricular (LV) remodelling, characterized by increased LV wall thickness, reduced end-diastolic diameter (EDD), and increased relative wall thickness (RWT) (Figure 2A–C). In males, mirabegron accentuated these structural changes, resulting in thicker ventricular walls, smaller LV chambers, and increased RWT compared to MHS-only mice. In contrast, females showed minimal response to mirabegron, with only a modest reduction in EDD. Direct comparisons between male and female responses for the principal echocardiographic parameters are presented in Supplementary Figure S2.

**Figure 3:**
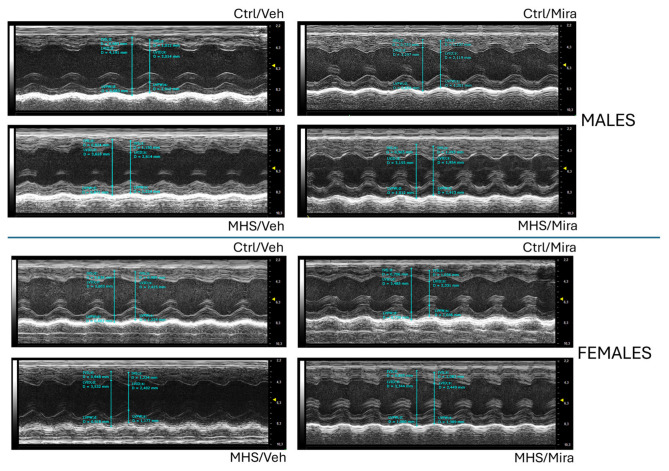
Representative M-mode echo LV tracings of control (Ctrl) and MHS male and female mice treated or not treated (Veh) with mirabegron (Mira).

Additional echocardiographic parameters are summarized in Table 2. In males, mirabegron treatment in the context of MHS was associated with reductions in interventricular septal thickness in diastole (IVSd) and end-systolic diameter (ESD), effects not observed in females. Representative M-mode echocardiographic images are shown in Figure 3. Using two-dimensional echocardiography and Simpson’s method, we found that end-diastolic (EDV) and end-systolic (ESV) LV volumes were significantly reduced in mirabegron-treated males, but not in females (Figure 2D,E). This was associated with a higher ejection fraction (EF) in males, while cardiac output (CO) remained unchanged across all groups.

**Figure 4:**
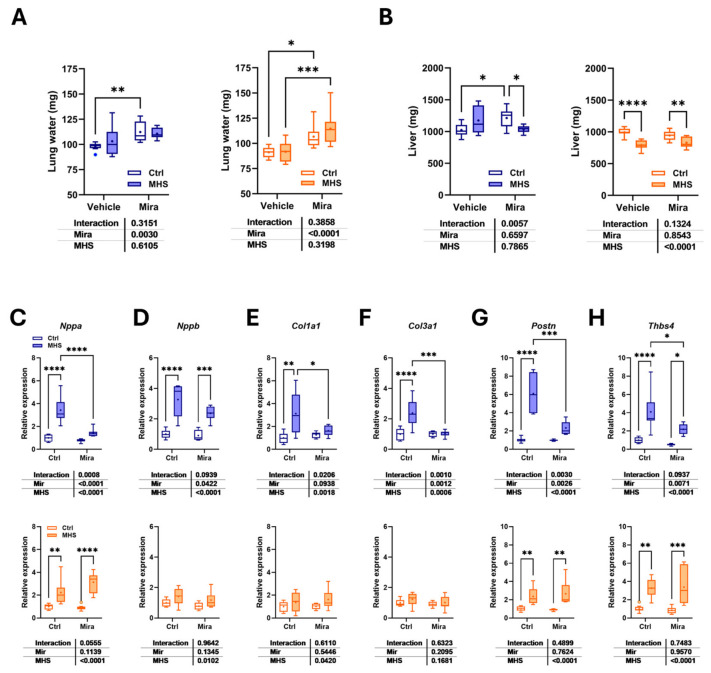
Mirabegron sex-dependent pattern on lungs, liver and pathological LV hypertrophy gene expression. (**A**) Lung water content (wet lungs weight − dry lungs weight). (**B**) Liver weight. Males: blue and left panel. Females: orange and right panel. LV gene expression in control and MHS treated with or without Mira. Males: blue and upper panel. Females: orange and the bottom panel. (**C**) Atrial natriuretic peptide (*Nppa*). (**D**) Brain natriuretic peptide (*Nppb*). (**E**) Collagen 1 alpha (*Col1a1*). (**F**) Collagen 3 alpha (*Col3a1*). (**G**) Periostin (*Postn*). (**H**) Thrombospondin 4 (*Thbs4*). Results are expressed as mean ± SEM, with two-way ANOVA followed by Fisher’s LSD test. The results of the two-way ANOVA are presented below the graph for each variable and the interaction. * *p* < 0.05, ** *p* < 0.01, *** *p* < 0.001 and **** *p* < 0.0001 between indicated groups (n = 7–8 mice/group).

Diastolic function parameters are reported in Table 3. In agreement with previous studies, 4 weeks of MHS did not induce overt pulmonary congestion, whereas longer exposure is required to increase lung water content (Figure 4A). Interestingly, mirabegron treatment increased lung water content in both control and MHS mice, although no additional increase was observed in MHS animals. Liver weight was increased by mirabegron in males but not in females. Conversely, MHS reduced liver weight in females, whereas in males, a similar reduction was observed only in mirabegron-treated animals (Figure 4B).

### 3.3. Effects on Cardiac Gene Expression

We next assessed the expression of genes associated with pathological cardiac remodelling. As expected, atrial natriuretic peptide (Nppa) expression was increased in MHS mice receiving vehicle. This increase persisted in females treated with mirabegron but was almost completely abolished in males (Figure 4C). Similarly, brain natriuretic peptide (Nppb) expression was elevated in both sexes following MHS, although the increase was less pronounced in females (Figure 4D).

Gene expression of extracellular matrix components revealed a marked sex-dependent pattern. In females, collagen genes (*Col1a1* and *Col3a1*) were only minimally affected by MHS, despite previously observed increases in myocardial fibrosis [8,9,17], and mirabegron had no additional effect. In contrast, MHS significantly increased collagen gene expression in males, an effect that was completely prevented by mirabegron (Figure 4E,F). Similarly, markers of fibroblast activation, including periostin (Postn) and thrombospondin-4 (Thbs4), were upregulated in vehicle-treated MHS mice and attenuated by mirabegron in males but not females (Figure 4G,H). Supplementary Figure S3 provides side-by-side comparisons of male and female molecular responses to mirabegron treatment.

### 3.4. The BAT Response to Mirabegron and the MHS

**Figure 5:**
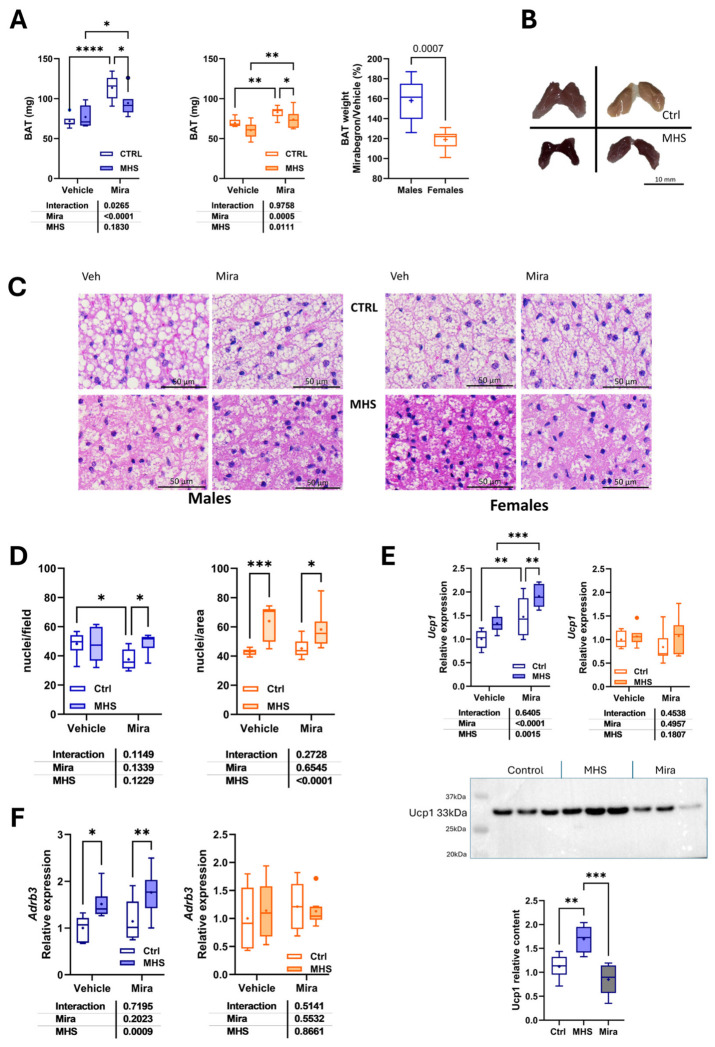
Effects of MHS and Mira on brown fat. (**A**) Brown adipose tissue (BAT) scapular depot weight. Right panel: increase in BAT weight in control mice treated with Mira. Males: blue and left panel. Females: orange and right panel. (**B**) Representative pictures of the BAT depot in two female Mira-treated control mice (top) and two MHS mice. Scale bar: 10 mm. (**C**) Hematoxylin/eosin-stained BAT sections from a mouse of each group. Scale bar: 50 µm. (**D**) Number of nuclei per microscope field. (**E**) *Ucp1* mRNA levels in the BAT and UCP1 protein content in the BAT in males. (**F**) *Adrb3* gene expression. Results are expressed as mean ± SEM. The results of the two-way ANOVA are presented below the graph for each variable and the interaction (MHS × Mira). * *p* < 0.05, ** *p* < 0.01, *** *p* < 0.001 and **** *p* < 0.0001 between indicated groups (n = 7–8 mice/group; 6 mice/group for the protein content).

We then examined the scapular brown adipose tissue (BAT) depot. Mirabegron increased BAT weight in control animals, with a more pronounced effect in males (Figure 5A). Interestingly, MHS partially blunted this effect. Representative BAT images (Figure 5B) revealed a darker appearance following MHS, consistent with reduced lipid content and increased thermogenic activation. Histological analysis confirmed a decrease in adipocyte size (Figure 5C), reflected by an increased number of nuclei per field (Figure 5D).

Activation of β3-adrenergic signalling is expected to enhance thermogenesis and induce expression of the mitochondrial uncoupling protein 1 (Ucp1). As anticipated, mirabegron increased Ucp1 mRNA levels, particularly in males (Figure 5E). In contrast, expression of the β3-adrenergic receptor gene (Adrb3) was not significantly affected by mirabegron in control mice or in females under MHS conditions. However, Adrb3 expression was elevated in MHS males regardless of treatment (Figure 5F). Direct male–female comparisons of BAT-related outcomes are presented in Supplementary Figure S4.

**Figure 6:**
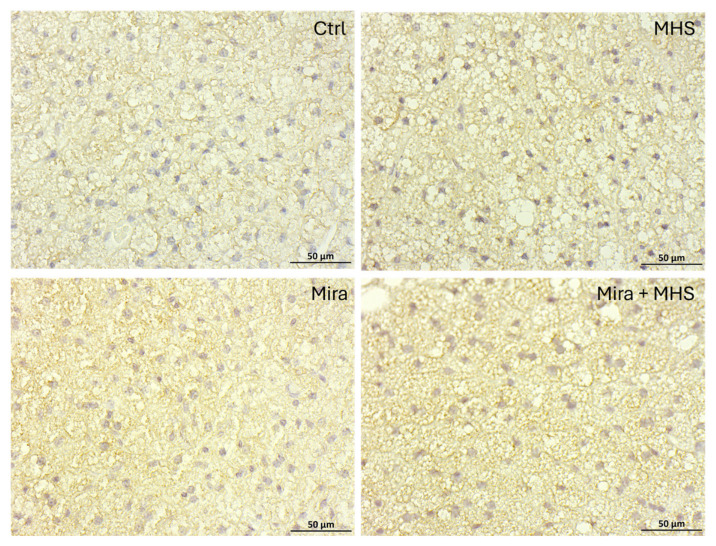
UCP1 immunohistological detection in female BAT sections. UCP1 protein is evenly distributed in the BAT (orange-brown coloration) and is more abundant in the section from mice receiving Mira and/or MHS. Bar scale: 50 µm.

Representative immunohistochemical analysis of BAT sections (Figure 6) showed increased UCP1 protein staining in animals exposed to mirabegron and/or MHS, supporting activation of the thermogenic programme.

### 3.5. Mirabegron Effects on Cardiomyocyte and Extracellular Matrix Remodelling After MHS

**Figure 7:**
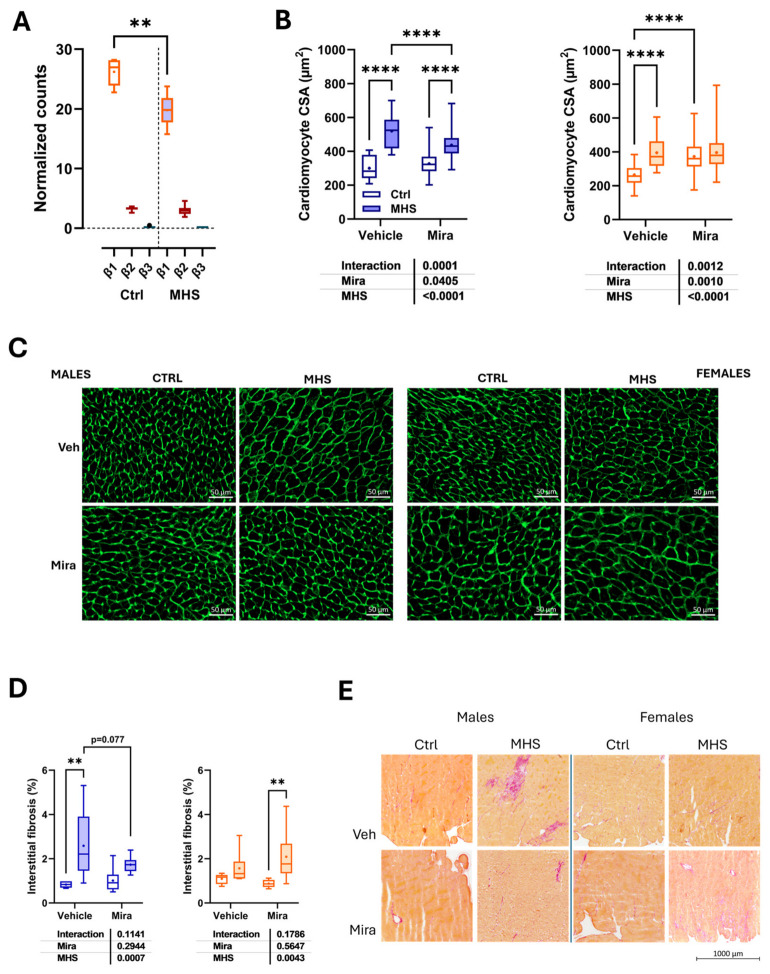
β-adrenergic receptor gene expression in the heart and myocardial morphological changes related to mirabegron treatment. (**A**) Beta-adrenergic receptor subtypes mRNA levels in control and MHS mice. Using bulk RNA sequencing data from the LV of young control and MHS mice (three males and three females per group; GEO accession number: GSE240171), we extracted the normalized counts of the three adrenergic receptors (Adrb1 or β1, Adrb2 or β2, and Adrb3 or β3) in control and MHS mice. (**B**) Cardiomyocyte cross-sectional area. (**C**) Representative images of wheat germ agglutinin-FITC staining from LV sections of the various indicated groups. (**D**) Myocardial interstitial fibrosis. (**E**) Representative images of picrosirius red staining of male and female LV sections. Results are expressed as mean ± SEM. The results of the two-way ANOVA are shown below each graph for each variable and the interaction (MHS × Mira). ** *p* < 0.01, and **** *p* < 0.0001 between the indicated groups (n = 7–8 mice per group).

The mouse myocardium exhibits minimal expression of the β3-adrenergic receptor (β3-AR). To confirm this in our model, we analyzed previously published left ventricular (LV) bulk RNA sequencing data [8; GEO accession number: GSE240171]. As shown in Figure 7A, normalized gene counts revealed that β3-AR (Adrb3) expression in the LV was virtually undetectable compared to β1-AR (Adrb1) and β2-AR (Adrb2) in both control and MHS mice (three males and three females per group). Notably, Adrb1 expression was reduced in MHS animals, whereas Adrb2 levels remained unchanged.

Cardiomyocyte size was assessed on LV sections using fluorescent wheat germ agglutinin (WGA) staining. As expected, MHS significantly increased cardiomyocyte cross-sectional area (CSA) in males, regardless of mirabegron treatment. However, CSA was significantly smaller in males receiving mirabegron, indicating a partial attenuation of hypertrophic remodelling (Figure 7B,C). In contrast, in females, mirabegron treatment increased cardiomyocyte size in control animals, whereas MHS did not further augment CSA, suggesting a distinct, sex-specific response.

Assessment of extracellular matrix remodelling showed that MHS increased interstitial fibrosis in both males and females (Figure 7D,E). In males, mirabegron treatment was associated with a trend toward reduced fibrosis, although this effect did not reach statistical significance (*p* = 0.077).

Together, these findings indicate that mirabegron modulates cardiomyocyte hypertrophy and extracellular matrix remodelling in a sex-dependent manner. Importantly, given the absence of detectable β3-AR expression in the myocardium, these cardiac effects are likely mediated indirectly.

## 4. Discussion

The present study demonstrates that pharmacological activation of β3-adrenergic signalling with mirabegron induces sex-dependent responses in a murine HFpEF-like model generated by combined metabolic and hypertensive stress (MHS). Overall, mirabegron attenuated several indices of cardiac remodelling in male mice, whereas its effects were considerably less pronounced in females. These findings further emphasize the importance of considering biological sex as a critical determinant of disease progression and therapeutic responsiveness in HFpEF-like conditions.

HFpEF is increasingly recognized as a systemic syndrome involving complex interactions among cardiovascular, metabolic, inflammatory, and neurohumoral pathways rather than a disorder confined to the myocardium alone [1,5]. Accordingly, growing attention has focused on extracardiac contributors to disease progression, including adipose tissue. We previously demonstrated that our MHS model reproduces several important features of HFpEF, including cardiac hypertrophy, concentric ventricular remodelling, left atrial enlargement, myocardial fibrosis, reduced exercise capacity, and preserved ejection fraction [8]. Furthermore, we and others have reported important sex-dependent differences in disease severity and progression in experimental heart failure models [8,9,16,17]. However, as with all animal models, the present model should be viewed as HFpEF-like rather than a complete reproduction of the human syndrome, given its relatively short duration and its inability to fully capture the complexity and heterogeneity observed in patients [4,5,6,7].

Adipose tissue is now widely recognized as an active endocrine organ capable of influencing distant tissues through the secretion of bioactive molecules, extracellular vesicles, and regulatory RNAs [18,19]. While white adipose tissue has traditionally received the greatest attention, brown adipose tissue (BAT) has emerged as an important regulator of systemic metabolism and cardiovascular homeostasis [20,21]. Beyond its classical role in thermogenesis, BAT secretes numerous factors, collectively termed batokines, that modulate inflammation, energy metabolism, vascular function, and cardiac remodelling [20,21].

The original hypothesis underlying this study was that activation of β3-adrenergic receptors in BAT could influence cardiac remodelling during the development of HFpEF. Consistent with this hypothesis, mirabegron increased BAT mass and enhanced Ucp1 expression, particularly in males. This enhanced BAT response was associated with reduced cardiac hypertrophy, attenuation of left atrial enlargement, lower expression of hypertrophic and fibrotic markers, and modest improvements in ventricular structure. Collectively, these observations support an association between BAT activation and improved cardiac remodelling. However, these mechanistic interpretations remain exploratory and hypothesis-generating. The present study was not designed to establish causal links between BAT activation and cardiac protection, and alternative systemic mechanisms may account for some of the observed effects. However, the present experiments do not directly establish causality, and BAT activation should therefore be regarded as a plausible contributor rather than a proven mediator of the cardioprotective effects of mirabegron. Additional endocrine, autonomic, hemodynamic, and systemic metabolic mechanisms may also participate in the observed responses.

Several observations support the possibility that indirect mechanisms predominate in our model. First, analysis of previously generated LV RNA-sequencing data revealed virtually undetectable levels of Adrb3 expression in the myocardium compared with Adrb1 and Adrb2. Second, the magnitude of BAT activation paralleled the cardiac response across sexes. Third, previous studies have demonstrated that β3-adrenergic receptor activation in adipocytes can influence cardiac remodelling via endocrine signalling pathways. For example, Lin et al. reported that BAT-specific β3-adrenergic signalling attenuates AngII-induced cardiac remodelling by suppressing exosomal release of inducible nitric oxide synthase from BAT [13]. Other BAT-derived factors, including FGF21 and 12,13-diHOME, have also been implicated in cardiometabolic protection [20,22,23,]. Nevertheless, because we did not directly assess circulating batokines or experimentally manipulate BAT, our results cannot distinguish BAT-mediated effects from other systemic consequences of β3-adrenergic stimulation.

An important finding of the present study is the markedly different response observed between males and females. Biological sex is increasingly recognized as a major determinant of cardiovascular remodelling and heart failure progression. Consistent with our observations, Kala and colleagues demonstrated in Ren-2 transgenic rat models that sex significantly influences ventricular remodelling, myocardial fibrosis, renal dysfunction, and progression toward heart failure. Moreover, therapeutic responses differed between males and females, emphasizing the importance of considering sex as a biological variable in experimental cardiovascular research [24,25]. Our findings, therefore, extend previous observations from other experimental models to a murine HFpEF-like context. Direct comparisons between sexes are provided in Supplementary Figures S1–S4 and illustrate the overall tendency toward a more pronounced cardiac and BAT response pattern in males than in females. Importantly, although several outcomes exhibited qualitatively different response patterns between males and females, formal sex-by-treatment interactions were not significant for all variables. Consequently, observed sex differences should be interpreted within the context of the statistical interaction analyses and confirmed in larger cohorts.

The mechanisms responsible for the attenuated female response observed here remain uncertain. Differences in BAT biology likely contribute, as numerous studies have reported sex-dependent regulation of thermogenic activity, mitochondrial function, and β-adrenergic responsiveness [26,27,28,29,30,31,32,33,34]. Estrogens can modulate BAT activity through both central and peripheral pathways, including hypothalamic AMPK signalling and direct regulation of thermogenic genes such as Ucp1 [30,31]. However, BAT activation alone is unlikely to explain the observed dimorphism fully. Additional mechanisms may include sex-specific differences in adrenergic receptor signalling, mitochondrial substrate utilization, oxidative metabolism, inflammatory responses, and neurohumoral activation. All of these processes have been implicated in the pathophysiology of HFpEF and may influence responsiveness to β3-adrenergic stimulation [1,5].

Although several findings support the beneficial effects of mirabegron in males, the overall response was not uniformly favourable across all endpoints. For example, mirabegron increased relative wall thickness, elevated heart rate in MHS males, and was associated with increased lung water content in both control and MHS animals. Changes in liver weight were also complex and differed between sexes. These observations suggest that β3-adrenergic receptor activation induces multifaceted physiological adaptations whose overall consequences may vary depending on tissue, sex, and disease context. Therefore, interpretation of mirabegron as a purely cardioprotective intervention would likely oversimplify its biological effects.

The relationship between BAT activation and cardiac remodelling remains complex and may depend on the nature of the stimulus. In a previous study, we demonstrated that thermoneutral housing, a condition associated with markedly reduced BAT activity, attenuated several manifestations of the HFpEF-like phenotype [15]. At first glance, this observation appears difficult to reconcile with the beneficial effects associated with mirabegron-induced BAT activation observed here. One possible explanation is that basal and pharmacological BAT activation engage distinct mechanisms. Alternatively, mild environmental cold stress present under standard housing conditions may prime BAT responsiveness, allowing selective β3-adrenergic stimulation to activate adaptive pathways that differ from those engaged during chronic thermogenic stress. These observations further emphasize the context-dependent nature of BAT-mediated cardiovascular effects.

Clinical translation of these findings also warrants careful consideration. Although β3-adrenergic receptor agonists, including mirabegron, have generated substantial interest as potential cardiovascular therapies [11,12], clinical trials performed in patients with structural heart disease, chronic heart failure, or pulmonary hypertension have generally produced neutral or modest results [35,36,37,38]. Furthermore, BAT thermogenesis in humans appears to depend predominantly on β2-adrenergic rather than β3-adrenergic signalling [39], potentially limiting direct extrapolation of rodent findings to humans. Nevertheless, the broader concept that adipose tissue influences cardiac remodelling remains highly relevant, particularly in obesity-associated HFpEF, where adipose dysfunction is increasingly recognized as a key pathogenic factor [18,19,20,21].

Importantly, the sex-dependent effects observed in the present study may have translational implications. Contemporary cardiovascular research increasingly recognizes the need to incorporate sex as a biological variable in both preclinical and clinical investigations. Our findings suggest that future studies evaluating mirabegron, BAT-targeted therapies, or other adipose tissue-directed interventions should systematically include sex-stratified analyses, as treatment responsiveness may differ substantially between males and females. Such an approach may ultimately improve patient selection and facilitate the development of more personalized therapeutic strategies for HFpEF.

In summary, activation of the β3-adrenergic receptor by mirabegron was associated with sex-dependent modulation of cardiac remodelling in a murine HFpEF-like model. The preferential response observed in males coincided with greater BAT activation and reduced expression of pathological remodelling markers. While these findings are consistent with a contribution of BAT-derived signalling to cardiac protection, direct causality remains to be established. Future studies incorporating BAT-specific interventions, assessment of circulating batokines, and detailed characterization of sex-dependent signalling pathways will be required to clarify the mechanisms linking β3-adrenergic activation, adipose tissue function, and cardiac remodelling.

## 5. Study Limitations

Several limitations of this study should be acknowledged.

First, mirabegron treatment was initiated concurrently with the induction of metabolic and hypertensive stress (MHS). Consequently, our findings primarily address the effects of β3-adrenergic receptor activation on disease development and do not establish whether mirabegron would be effective as a therapeutic intervention in established HFpEF-like disease.

Second, although our results show a clear association between mirabegron-induced BAT activation and improvements in several indices of cardiac remodelling, the present study does not establish a causal relationship between these observations. Direct demonstration of BAT dependency would require dedicated approaches such as BAT ablation, denervation, transplantation, or genetic models targeting BAT function. Furthermore, we did not assess circulating batokines, extracellular vesicles, or autonomic mechanisms that could contribute to the observed cardiac responses. Therefore, BAT activation should be considered a plausible contributor rather than a proven mediator of the cardioprotective effects associated with mirabegron.

Third, we did not investigate downstream β3-adrenergic signalling pathways in BAT or other tissues, nor did we evaluate potential endocrine mediators such as FGF21 or 12,13-diHOME. As a result, the molecular mechanisms linking β3-adrenergic activation to cardiac remodelling remain incompletely characterized.

Fourth, the relatively small group sizes (n = 7–8 per group), combined with the mortality observed in a subset of MHS males, may have limited the statistical power to detect subtle treatment effects or sex-treatment interactions, particularly for fibrosis measurements and selected echocardiographic parameters. In addition, the exploratory nature of the study and the number of endpoints analyzed increase the possibility of type I statistical error. Therefore, some findings should be interpreted cautiously until confirmed in independent cohorts. Accordingly, findings derived from secondary or exploratory endpoints should be considered preliminary and interpreted with appropriate caution until replicated in adequately powered studies.

Fifth, our molecular analyses were limited primarily to mRNA measurements. Although these markers are widely used indicators of pathological remodelling, transcript abundance does not necessarily predict changes in protein expression or biological activity.

Sixth, metabolic parameters such as glucose homeostasis, insulin sensitivity, lipid metabolism, blood pressure, energy expenditure, and sympathetic nervous system activity were not assessed. Consequently, we cannot exclude the possibility that systemic metabolic or hemodynamic adaptations contributed to the observed cardiac effects.

Finally, although the combined AngII/high-fat diet model reproduces several important characteristics of HFpEF, including cardiac hypertrophy, concentric remodelling, left atrial enlargement, myocardial fibrosis, and preserved ejection fraction, it remains an HFpEF-like model. It cannot fully capture the complexity and heterogeneity of human HFpEF. Alternative models, such as HFD plus L-NAME [3], are widely employed in HFpEF research; each model captures different aspects of the syndrome. The AngII/HFD model was selected because it reliably reproduces several HFpEF-like features in both sexes, thereby allowing evaluation of sex-dependent responses to therapeutic interventions.

## 6. Conclusions

Activation of β3-adrenergic signalling with mirabegron induces sex-dependent effects in a murine model of HFpEF. The more pronounced response observed in males was associated with greater BAT activation and reduced pathological cardiac remodelling, although causality remains to be established. These findings highlight the importance of considering sex as a biological variable and support further investigation into adipose tissue–heart interactions as potential therapeutic targets in HFpEF.

## Figures and Tables

**Table 1:** Sequences of primers used in this study.

Symbol	Description	Forward Sequence Reverse Sequence
*Col1a1*	Collagen Type I Alpha 1 Chain	5′-CAT TGT GTA TGC AGC TGA CTT C-3′ 5′CGC AAA CAC TCT ACA TGT CTA GG-3′
*Col3a1*	Collagen Type III Alpha 1 Chain	5′-TCT CTA GAC TCA TAG GAC TGA CC-3′ 5′ TTC TTC TCA CCC TTC TTC ATC C-3′
*Nppa*	Natriuretic Peptide B	5′-CTC CTT GGC TGT TAT CTT CGG-3′ 5′-GGG TAG GAT TGA CAG GAT TGG-3′
*Nppb*	Natriuretic Peptide A	5′-AGG TGA CAC ATA TCT CAA GCT G-3′ 5′-CTT CCT ACA ACA TCA GTG C-3′
*Ppia*	Cyclophilin A	5′-TTC ACC TTC CCA AAG ACC AC-3′ 5′-CAA ACA CAA ACG GTT CCC AG-3′
*Postn*	Periostin	5′-GCT TTC GAG AAA CTG CCA CG-3′ 5′-ATG GTC TCA AAC ACG GCT CC-3′
*Thbs4*	Thrombospondin 4	5′-GAT ACT GAC GGG GAT GGG AG-3′ 5′-CGT CAC TGT CTT GGT TGG TG-3′
*Ucp1*	Uncoupled protein 1	5′-GCT TCT ACG ACT CAG TCC AA-3′ 5′-CTC TGG GCT TGC ATT CTG AC-3′
*Adrb3*	Adrenoceptor Beta 3	5′-AGG AAG CTT GCT TGA TCC-3′ 5′-AGA GAG AGA GGA CGG TGA AAC-3′
*Rpl13*	Ribosomal Protein L13	5′-CGG CTG AAG CCT ACC AGA AA-3′ 5′-GGA GTC CGT TGG TCT TGA GG-3′

**Table 2:** Echo data in male and female mice after MHS and/or Mirabegron (Mira). Controls (C). Vehicle (Veh). Echo exams as described in the Methods section. PWd: diastolic posterior wall thickness; IVSd: diastolic interventricular septum; ESD: end-systolic LV diameter; LVM: LV mass; SV: stroke volume; HR: heart rate; CO: cardiac output. Results are expressed as the mean ± standard error of the mean (SEM). *p*-values were calculated using Fisher’s LSD test after a two-way ANOVA. Against respective control (Ctrl) group: a: *p* < 0.05; b: *p* < 0.01; c: *p* < 0.001; d: *p* < 0.0001. Against respective Veh group: e: *p* < 0.05; f: *p* < 0.01; g: *p* < 0.001; h: *p* < 0.0001.

Males							
Parameters	Ctrl/Veh (n = 8)	MHS/Veh (n = 8)	Ctrl/Mira (n = 8)	MHS/Mira (n = 7)	MHS	Mira	MHS × M
PWd, mm	0.79 ± 0.016	1.03 ± 0.027 d	0.75 ± 0.014	1.05 ± 0.021 d	<0.0001	0.52	0.087
IVSd, mm	0.75 ± 0.013	0.91 ± 0.023 d	0.75 ± 0.013	0.99 ± 0.020 d,g	<0.0001	0.032	0.028
ESD, mm	2.79 ± 0.075	2.77 ± 0.098	2.40 ± 0.085 f	2.01 ± 0.071 b,h	<0.0001	0.026	0.034
LVM, mg	106 ± 1.8	134 ± 5.1 d	100 ± 2.5	123 ± 5.2 c	<0.0001	0.046	0.57
SV, mm	31 ± 2.2	31 ± 1.5	31 ± 1.7	26 ± 1.3	0.22	0.27	0.22
HR, bpm	498 ± 10.4	513 ± 17.0	518 ± 14.0	561 ± 11.7 a,e	0.044	0.021	0.31
CO, ml/min	15.2 ± 1.07	15.8 ± 1.05	15.9 ± 0.60	14.6 ± 1.22	0.71	0.81	0.37
**Females**							
Parameters	**Ctrl/Veh (n = 8)**	**MHS/Veh (n = 8)**	**Ctrl/Mira** **(n = 8)**	**MHS/Mira** **(n = 8)**	**MHS**	**Mira**	**MHS × M**
PWd, mm	0.71 ± 0.024	0.88 ± 0.024 b	0.71 ± 0.011	0.96 ± 0.056 c	<0.0001	0.23	0.27
IVSd, mm	0.70 ± 0.020	0.84 ± 0.015 d	0.67 ± 0.010	0.81 ± 0.031 d	<0.0001	0.15	0.90
ESD, mm	2.52 ± 0.105	2.33 ± 0.061	2.34 ± 0.096	2.20 ± 0.076	0.0680	0.088	0.78
LVM, mg	88 ± 1.7	109 ± 3.2 d	81 ± 2.9	103 ± 4.0 d	<0.0001	0.044	0.83
SV, mm	31 ± 0.7	30 ± 1.5	25 ± 0.7 g	26 ± 1.3 e	0.62	0.0022	0.26
HR, bpm	516 ± 11.2	528 ± 6.0	514 ± 15.0	538 ± 17.6	0.19	0.74	0.67
CO, ml/min	15.4 ± 0.85	15.6 ± 0.84	13.1 ± 0.43 e	14.0 ± 0.46	0.40	0.0061	0.64

**Table 3:** Echo diastolic parameters in male and female mice after MHS and/or Mirabegron (Mira). Controls (C). Vehicle (Veh). Echo exams as described in the Methods section. E, wave velocity; A, wave velocity; E, wave slope; IVRT, isovolumetric relaxation time; E’, wave velocity; A’, wave velocity; LA, diameter. Results are expressed as the mean ± standard error of the mean (SEM). *p*-values were calculated using Fisher’s LSD test after a two-way ANOVA. Against respective control group: a: *p* < 0.05; b: *p* < 0.01; c: *p* < 0.001; d: *p* < 0.0001. Against respective Veh group: e: *p* < 0.05; f: *p* < 0.01; g: *p* < 0.001.

Males							
Parameters	Ctrl/Veh (n = 8)	MHS/Veh (n = 8)	Ctrl/Mira (n = 8)	MHS/Mira (n = 7)	MHS	Mira	MHS × M
E, mm/s	659 ± 14.8	537 ± 21.0 c	653 ± 23.7	533 ± 21.2 c	<0.0001	0.81	0.97
A, mm/s	431 ± 16.5	388 ± 21.1	391 ± 13.5	405 ± 19.9	0.43	0.49	0.12
E slope	−39,160 ± 2804	−33,681 ± 1799	−37,444 ± 1566	−33,247 ± 2466	0.037	0.63	0.77
IVRT, ms	16.8 ± 0.53	16.0 ± 0.35	15.7 ± 0.49	15.3 ± 0.47	0.24	0.48	0.80
E’, mm/s	29.4 ± 0.99	24.0 ± 1.90 b	30.0 ± 0.77	22.8 ± 0.75 c	<0.0001	0.82	0.50
A’, mm/s	17.8 ± 0.81	16.5 ± 1.29	14.9 ± 0.44 e	14.8 ± 0.96	0.46	0.021	0.55
E/A	1.5 ± 0.04	1.4 ± 0.03 a	1.7 ± 0.05 e	1.3 ± 0.05 d	<0.0001	0.40	0.020
E/E’	20.8 ± 1.17	21.1 ± 0.90	21.9 ± 0.94	23.7 ± 1.20	0.32	0.96	0.58
LA diam, mm	2.34 ± 0.049	2.46 ± 0.081	2.18 ± 0.041	2.42 ± 0.067 b	0.0058	0.11	0.32
**Females**							
Parameters	**Ctrl/Veh (n = 8)**	**MHS/Veh (n = 8)**	**Ctrl/Mira (n = 8)**	**MHS/Mira (n = 8)**	**MHS**	**Mira**	**MHS × M**
E, mm/s	560 ± 23.9	604 ± 28.9	532 ± 11.3	650 ± 45.2 b	0.012	0.76	0.22
A, mm/s	374 ± 11.8	398 ± 21.8	315 ± 7.2	302 ± 22.3	0.74	<0.0001	0.29
E slope	−38,869 ± 1323	−35,653 ± 2291	−36,251 ± 1234	−39,024 ± 4731	0.89	0.94	0.29
IVRT, ms	15.1 ± 0.29	16.1 ± 0.57	15.9 ± 0.47	16.0 ± 0.43	0.44	0.29	0.32
E’, mm/s	30.5 ± 0.29	24.1 ± 1.10 c	29.6 ± 0.67	26.5 ± 1.20 a	<0.0001	0.14	0.33
A’, mm/s	18.5 ± 0.78	17.8 ± 0.67	16.0 ± 0.43 e	13.0 ± 1.31 a,f	0.029	0.0003	0.31
E/A	1.6 ± 0.04	1.5 ± 0.07	1.7 ± 0.06	2.2 ± 0.20 b,g	0.0004	0.018	0.031
E/E’	18.6 ± 0.71	23.1 ± 1.23 b	18.1 ± 0.65	24.6 ± 1.15 c	<0.0001	0.20	0.43
LA diam, mm	2.13 ± 0.0027	2.30 ± 0.053 a	2.13 ± 0.055	2.38 ± 0.088 b	0.0017	0.48	0.49

## Data Availability

The authors will make the raw data supporting this article’s conclusions available upon request.

## Supplementary Materials

The following supporting information can be downloaded at: https://www.mdpi.com/article/10.3390/biomedicines14071633/s1, **Figure S1**. Sex-dependent effects of mirabegron on cardiac morphology and remodelling. Direct comparison of male and female mice subjected to metabolic and hypertensive stress (MHS) and treated or not with mirabegron (Mira). Data are presented to facilitate visualization of sex-dependent response patterns. Results are expressed as mean ± SEM of the ratio of MHS mice heart weight over the mean heart weight of control mice. Statistical analyses were performed as described in the Methods section. Males are shown in blue and females in orange. **Figure S2. **Sex comparison of echocardiographic responses to mirabegron treatment. Comparison of the effects of mirabegron on key echocardiographic parameters in male and female mice exposed to MHS over controls. The figure highlights differences in ventricular volumes and function between sexes following β3-adrenergic receptor activation in MHS animals compared to controls. Results are expressed as mean ± SEM. Statistical analyses were performed as described in the Methods section. **Figure S3. **Sex-dependent regulation of cardiac remodelling genes following mirabegron treatment. Comparison of left ventricular gene expression profiles in male and female mice after MHS and mirabegron treatment. Expression levels of hypertrophic and fibrotic markers are presented to facilitate direct visualization of sex-dependent molecular responses. Results are expressed as mean ± SEM. Statistical analyses were performed as described in the Methods section. **Figure S4. **Comparison of BAT responses to mirabegron in males and females. Brown adipose tissue (BAT) responses to β3-adrenergic receptor activation in male and female mice. BAT mass, thermogenic markers, and related parameters are shown to illustrate sex-dependent differences in BAT responsiveness. Results are expressed as mean ± SEM. Statistical analyses were performed as described in the Methods section.

## Abbreviations

The following abbreviations are used in this manuscript:

HFpEF	Heart failure with preserved ejection fraction
AngII	Angiotensin II
MHS	Metabolic and hypertensive stress (AngII + HFD)
HFD	High-fat diet
LA	Left atrial or left atrium
LV	Left ventricle
CH	Cardiac hypertrophy
EF	Ejection fraction
UCP1	Uncoupled protein 1
ECM	Extracellular matrix
EDD	End-diastolic diameter
ESD	End-systolic diameter
RWT	Relative wall thickness
SV	Stroke volume
CO	Cardiac output
BW	Body weight
CSA	Cross-sectional area
L-NAME	L-NG-Nitroarginine Methyl Ester
BP	Blood pressure
EDV	End-diastolic volume
ESV	End-systolic volume
Nppa	Atrial natriuretic peptide
Nppb	Brain natriuretic peptide
Col1a	Pro-collagen1 alpha
Col3a	Pro-collagen3 alpha
Postn	Periostin
Thbs4	Thrombospondin 4
PW	Posterior wall
IVSW	Interventricular septal wall
SEM	Standard error of the mean
RWT	Relative LV wall thickness
Ppia	Cyclophilin A
RPL13	L13 ribosomal protein
iNOS	Nitric oxide synthase, inducible
β3-AR	Adrenergic receptor, beta 3
BAT	Brown adipose tissue
RAAS	Renin–angiotensin–aldosterone system
SNS	Sympathetic nervous system
