# Supplementary material for: Sex-Dependent Cardiac Responses to β3-Adrenergic Receptor Activation in a Murine Model of Heart Failure with Preserved Ejection Fraction
Source: Biomedicines. 2026 Jul 20;14(7):1633. doi: 10.3390/biomedicines14071633 (PMC13406397; doi:10.3390/biomedicines14071633)
Supplement: Supplementary file 1 [file biomedicines-14-01633-s001.zip › biomedicines-4425390-supplementary.pdf]

Supplemental data

Sex-dependent cardiac responses to β3-adrenergic receptor activation in a murine model of heart failure with preserved ejection fraction.

Sara-Ève Thibodeau 1,2,3, Élisabeth Walsh-Wilkinson 1,2,3, Emylie-Ann Labbé 1,2,3, Diwaba Carmel Teou 1,2,3, Ma-rie-Lune Legros 1,2,3, Audrey Morin-Grandmont 1,2,3, Jacques Couet 1,2,3

1 Département de Médecine, Faculté de Médecine, Université Laval, Québec City, QC G1V 0A6, Canada;

sara-eve.thibodeau@criucpq.ulaval.ca (S.-È.T.); elisabeth.walsh-wilkinson.1@ulaval.ca (É.W.-W.); emylie-ann.labbe.1@ulaval.ca (E.-A.L.); diwaba-carmel.teou.1@ulaval.ca (D.C.T.); ma-rie-lune.legros.1@ulaval.ca (M.-L.L.); audrey.morin-grandmont@criucpq.ulaval.ca

2 Groupe de Recherche sur les Valvulopathies, Centre de Recherche de l'Institut Universitaire de Cardiologie et de Pneumologie de Québec, Université Laval, Québec City, QC G1V 4G5, Canada

3: Centre de recherche de l'Institut universitaire de cardiologie et de pneumologie de Québec, Université Laval, Québec City, QC, Canada

Correspondence: Jacques.couet@med.ulaval.ca (J.C.)

Supplementary Figures S1–S4 provide direct comparisons between males and females of the principal cardiac and BAT-related outcomes to facilitate visualization of sex-dependent response patterns.

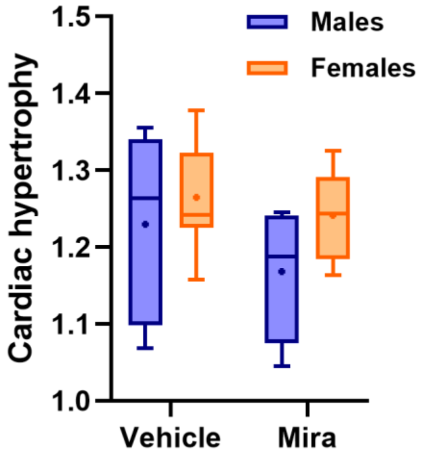

**Interaction**	**0.5283**
**Mira**	**0.1707**
**Sex**	**0.0838**

Figure S1. Sex-dependent effects of mirabegron on cardiac morphology and remodelling.

Direct comparison of male and female mice subjected to metabolic and hypertensive stress (MHS) and treated or not with mirabegron (Mira). Data are presented to facilitate visualization of sex-dependent response patterns. Results are expressed as mean ± SEM of the ratio of MHS mice heart weight over the mean heart weight of control mice. Statistical analyses were performed as described in the Methods section. Males are shown in blue and females in orange.

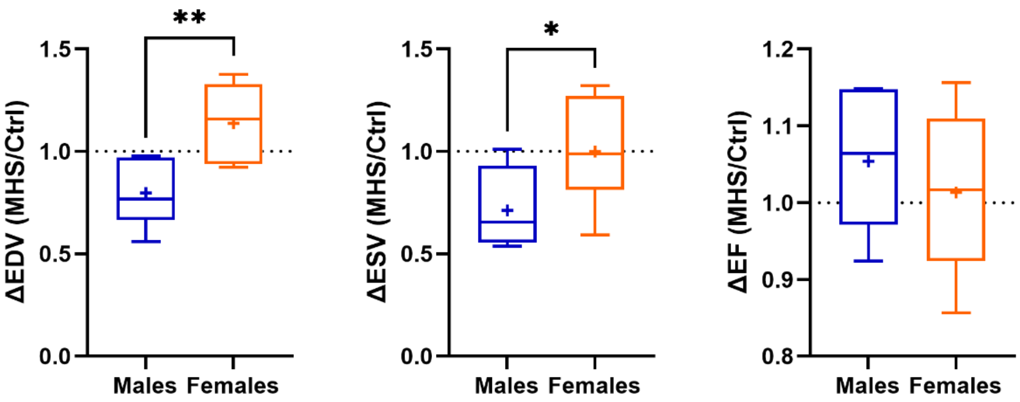

Figure S2. Sex comparison of echocardiographic responses to mirabegron treatment.

Comparison of the effects of mirabegron on key echocardiographic parameters in male and female mice exposed to MHS over controls. The figure highlights differences in ventricular volumes and function between sexes following β3-adrenergic receptor activation in MHS animals compared to controls. Results are expressed as mean ± SEM. Statistical analyses were performed as described in the Methods section.

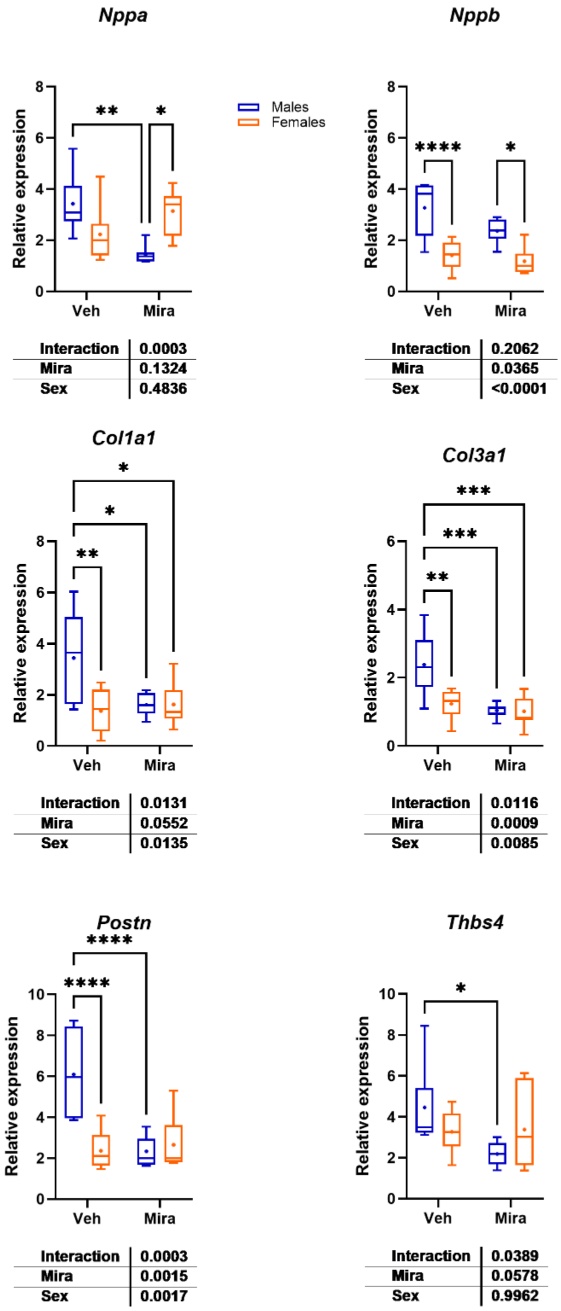

Figure S3. Sex-dependent regulation of cardiac remodelling genes following mirabegron treatment**.**

Comparison of left ventricular gene expression profiles in male and female mice after MHS and mirabegron treatment. Expression levels of hypertrophic and fibrotic markers are presented to facilitate direct visualization of sex-dependent molecular responses. Results are expressed as mean ± SEM. Statistical analyses were performed as described in the Methods section.

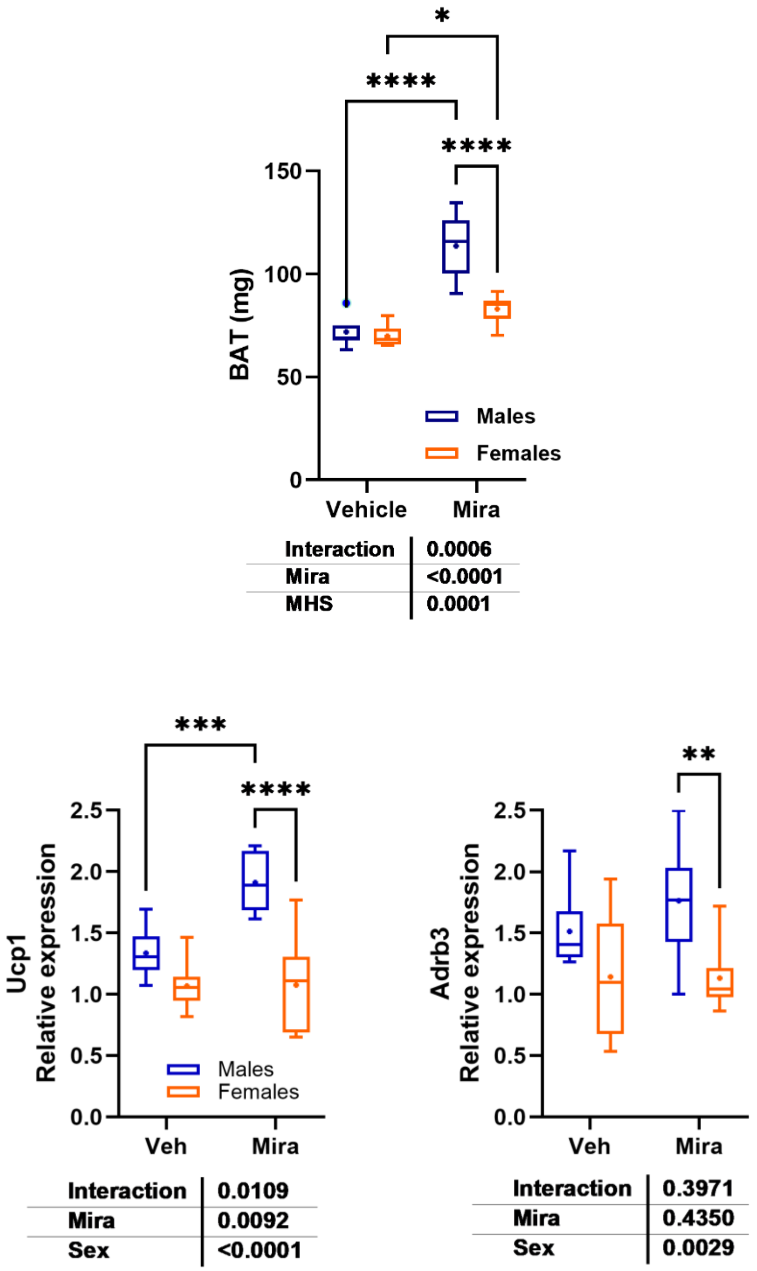

Figure S4. Comparison of BAT responses to mirabegron in males and females**.**

Brown adipose tissue (BAT) responses to β3-adrenergic receptor activation in male and female mice. BAT mass, thermogenic markers, and related parameters are shown to illustrate sex-dependent differences in BAT responsiveness. Results are expressed as mean ± SEM. Statistical analyses were performed as described in the Methods section.
